# Coregulation of HIV-1 dependency factors in individuals heterozygous to the CCR5-delta32 deletion

**DOI:** 10.1186/1742-6405-10-26

**Published:** 2013-11-18

**Authors:** Gero Hütter, Christian Blüthgen, Martin Neumann, Mark Reinwald, Daniel Nowak, Harald Klüter

**Affiliations:** 1 Institute of Transfusion Medicine and Immunology, Medical Faculty Mannheim, Heidelberg University; German Red Cross Blood Service Baden-Württemberg- Hessen, Mannheim, Germany; 2 Cellex GmbH, Dresden, Germany; 3 Medical Department III (Hematology, Oncology), Charité Campus Benjamin Franklin, Berlin, Germany; 4 Medical Department III (Hematology, Oncology), University Medical Centre Mannheim, Heidelberg University, Heidelberg, Germany

**Keywords:** CCR5-delta32, HIV-1, Coexpression, TOP1, CXCR2, SREBF2, TAP

## Abstract

**Background:**

CCR5-delta32 heterozygous individuals are susceptible to HIV-1. However, it is not clear if there is a relevant protective effect against transmission and a beneficial effect in terms of HIV progression which cannot be attributed to CCR5 surface density alone. Therefore we investigated HIV-1 dependency factors (HDF) which might be differently regulated in CCR5 wild type (WT) and CCR5-delta32 heterozygous individuals.

**Methods:**

We examined CD34+ hematopoietic progenitor cells derived from bone marrow samples from 19 healthy volunteers, 12 individuals with CCR5 WT and 7 with heterozygous CCR5-delta32 deletion. Samples were analyzed using a global gene expression oligonucleotide microarray (HG-U133plus 2.0, Affymetrix Inc.).

**Results:**

A total of 205 genes were found with altered expression (3fold difference, present call rate of 75%, p < 0.05) and 7 of these had a connection to HIV-1 pathogenesis. In 4 genes: TOP1, CXCR2, SREBF2, and TAP we found a different regulation which was consistent with a supposed beneficial effect for CCR5-delta32 heterozygotes.

**Conclusion:**

The CCR5-delta32 deletion is associated with other HDFs in HIV-1 pathogenesis as a possible explanation for beneficial effects regarding the deletion leading to a variant expression profile in heterozygous carriers of this mutation.

## Background

The human immunodeficiency virus type 1 (HIV-1) genome encodes a rather small number of 15 proteins. However, during infection and interaction with the target cells, the virus exploits multiple proteins of the host for its replication. Alterations of expression or functional changes based on genetic variances of critical genes are called HIV-dependency factors (HDFs). They are required for infection or disease progression, influencing the efficacy of HIV-1 transmission, viral load, and collapse of the immune system. Understanding these HDFs might play a key role in identifying new therapeutics strategies against the infection [1].

One of the most prominent HDF is the association of HIV-1 and the chemokine receptor CCR5. In combination with CD4, CCR5 is required for internalization of the viral genome. Up to date, over 70 mutations have been described in the CCR5 gene including the intensively studied 32 base pair deletion (CCR5-delta32) that introduces a premature stop-codon into the CCR5 locus [2,3].

Interestingly, CCR5 is highly preferred from the virus and people with a homozygous CCR5-delta32 deletion gain a nearly complete protection from HIV-1 transmission. This is remarkable as CXCR4 is always expressed as an alternative coreceptor in CCR5-deficient individuals and most HIV positive patients harbor a small fraction of CXCR4 using strains (X4 type) [4]. Therefore, CCR5 deficient individuals are frequently exposed against X4 but hardly ever get infected. It is still not clear whether this observation could only be explained by a presumed selective disadvantage of X4 in the absence of CCR5 or whether there are additional effects of the CCR5 deletion causing the protection from X4 transmission.

In addition, a certain protective effect in heterozygous CCR5-delta32 carriers is also controversially discussed (Table 1). It is well known that CCR5 receptor density on the target cells determines the susceptibility against transmission and that CCR5-delta32 heterozygotes have a reduced receptor expression on the surface [5]. However, CCR5 is a dynamic receptor and several studies have shown that CCR5 density in delta32 heterozygotes could be similar to wild type individuals and therefore the CCR5 surface expression might not be the single protective factor [6,7].

**Table 1:** A selection of published surveys concerning possible clinical benefits (transmission or progression) of the CCR5-delta32 heterozygous genotype

***N=***	***Study population***	***Benefit tested***	***Significance***	***Reference***
1427	HIV + vs. sero-negative control group	Transmission	P = 0.012	[8]
30	ESN	Transmission	P = 0.04	[9]
54	ESN	Transmission	P = 0.05*	[10]
2605	Woman	Transmission	OR: 0.63 (95% CI 0.44-0.90)	[11]
2996	High risk groups	Transmission	Risk = 0.30 (95% CI: 0.08-0.97)	[12]
1252	MSM	Transmission	NS	[13]
1200	HIV + vs control	Transmission, progression to AIDS	Both significant	[14]
108	IDU	Progression to AIDS	NS	[15]
127	Children	Progression to AIDS	Significant (p = 0.008) Effect is abrogated in case of a SDF1-3′A genotype	[16]
1955	Hemophilia patients	Progression to AIDS	Significant	[17]
512	Mother-child	Transmission, progression to AIDS	NS, significant	[18]

These controversial data suggest that there might be additional factors beside the CCR5-delta32 genotype like SDF 1-3′ which contribute to the clinical outcome of transmission and infection. (ESN = exposed seronegative, MSM = men having sex with men, IDU = injecting drug users, CI = confidence interval, OR = odds ratio, NS = not significant) *only significant in heterosexuals.

Taken together, the mechanism of a near-perfect protection of complete CCR5 deficiency as well as the observation that HIV-infected CCR5-delta32 heterozygotes may have a slower progression to AIDS has to be more elucidated and might help identifying other key players contributing to HIV pathogenesis [13,17].

Therefore, in this work we analyzed the gene expression patterns of CCR5-delta32 deletion in comparison to wild type carriers, further, how the alteration change in expression might contribute to the reduced clinical susceptibility and progression of HIV disease observed in carriers of this mutation. This might improve the understanding of the connection between HIV pathogenesis and immune system interaction of the underlying host.

## Results and discussion

We screened CD34+ cells of 19 specimen contained within a blood donor bank and identified 12 healthy donors carrying wild type (WT) CCR5 while the remaining seven carried the CCR5-delta32 deletion allele heterozygously (Table 2). Gene expression analysis revealed 205 genes differentially expressed between WT and heterozygous carriers of CCR5-delta32 mutation. 96 were found to be overexpressed in heterozygotes (Additional file 1: Table S1) whereas the remaining 109 genes were found to be overexpressed in WT samples, respectively.

**Table 2:** Results and characteristics of analyzed specimen

	***Specimen***	***Gender***	***Age***	***CCR5 WT***	***CCR5-delta32 heterozygous***
1	N0014/05	m	19	+	
2	N0015/05	f	53	+	
3	N0023/05	m	24	+	
4	N0024/05	f	21	+	
5	N0031/05	m	25	+	
6	N0035/05	m	21		+
7	N0091/06	m	30	+	
8	N0094/06	m	20	+	
9	N0103/06	m	32	+	
10	N0106/06	m	23	+	
11	N0118/07	f	28		+
12	N0136/07	f	29		+
13	N0153/07	f	29		+
14	N0158/07	m	25		+
15	N0163/07	f	23		+
16	N0200/08	f	74	+	
17	N0204/08	f	85	+	
18	N0305/09	f	28		+
19	N0312/09	f	34	+	

CD34+ hematopoeitic progenitor cells for genotyping were obtained by bone marrow aspiration.

**Figure 1:**
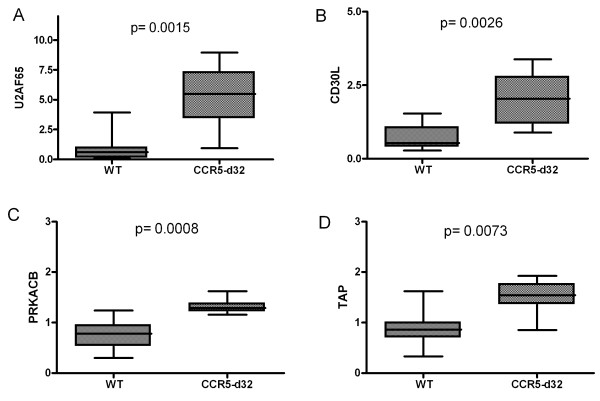
**Results of the micro array gene expression analysis of 19 specimens.** In the group of heterozygous CCR5-delta32 carriers 4 genes associated with HIV-1 pathogenesis have been found to be overexpressed: **A**. U2AF65, **B**. CD30L, **C**. PRKACB, and **D**. TAP. Normalized arbitrary expression levels.

**Figure 2:**
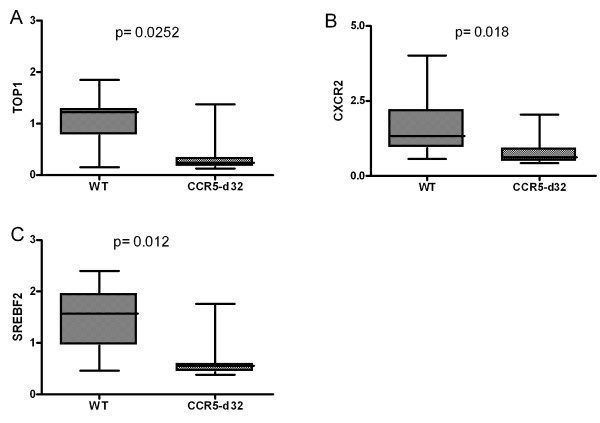
**Results of the micro array gene expression analysis of 19 specimens.** In wild type sample there were 3 genes associated with HIV-1 significantly overexpressed compared to heterozygous CCR5-delta32 carriers: **A**. TOP1, **B**. CXCR2, and **C**. SREBF2. Normalized arbitrary expression levels.

Further review of gene databases concerning the known or proposed function of these genes regarding HIV-1 infection revealed three genes in the WT group and four genes in the CCR5-delta32 group with significantly different overexpression profiles (Figures 1 and 2).

The theory that human host genes or polymorphic variants of genes may influence transmission or the outcome of HIV-1 infection goes back to the early 1980’s [19]. Improved techniques using high throughput assays provide insight into a detailed view in the pathogenesis of HIV-1 and possible host or HIV-dependency factors (HDF) [1]. Besides the well-characterized chemokine entry mechanism there are several other allelic mutations described in HDF genes such as IL10, IFNG, or KIR3DS1 (together with HLA-Bw4). In addition, several HLA subtypes have been found to be associated either with delayed or accelerated progression to AIDS [20].

However, some phenomena remain unsolved. Especially the role of the CCR5-delta32 deletion in transmission and maintaining the infection is under investigation and there are currently more open questions than answers. Recent progress in HIV gene therapy revealed new insights of the CCR5-delta32 deletion [21,22]. In the SB-728-T Sangamo trial with anti CCR5 zinc-finger nuclease (ZFN) treated lymphocytes in HIV patients, only one candidate with a CCR5-delta32 heterozygous genotype in this group achieved spontaneous virus control during HAART discontinuation. Taken into account that the CCR5 density in heterozygotes does not substantially differ from the density in wild types, it is astonishing that an additional knock down of less than a few percent of CCR5 expression by ZFNs has subsequently such a deep impact on viral replication. Consequently it was not surprising that Sangamo initiated a follow up trial especially for carriers of the CCR5-delta32 genotype (SB-728-902-Cohort 5) [23].

Most recently, the report of a long term control of HIV in two patients receiving an allogeneic stem cell transplantation (SCT) with a CCR5 wild type allo-graft (“Boston patients”) has brought up new questions. These patients showed a profound reduction of the viral reservoir, and declining HIV antibody levels with ongoing engraftment during the follow-up period of several years similar to the “Berlin patient” [24]. Finally, in 2013 antiretroviral medication was discontinued in these patients and none of them developed a rebound of HIV infection. Transplantation has led to a replacement of the old immune system, antiretroviral therapy has protected the new engrafting cells from re-infection, and probably GvHD has cleared residual viral reservoirs in the host. Interestingly, both patients were heterozygous for the CCR5-delta32 deletion [25].

### Genes with higher expression in the wild type group

From 109 genes significantly overexpressed in the wild type group, three were recognized to have an association with HIV-1 (Table 3). Of these three, CXCR2 is expressed in higher quantities in the wild type group. Like CXCR1, CXCR2 acts as a receptor for interleukin 8 (IL-8) and is differentially expressed in subsets of lymphocytes like CD56+/CD16+ NK-cells [26]. Both receptors share intracellular cross-talk and in cells expressing either CXCR1 or CXCR2, exposure to IL-8 leads to an internalization of these receptors. This effect is significant lower in cells expressing both of the receptors like the CD56+/CD16+ NK-cells [27]. Additionally, binding of IL-8 on the CXCR1 receptors causes an additional cross-phosphorylation of both, CCR5 and CXCR4, resulting in a decreased susceptibility against HIV-1. Only the association of IL-8/CXCR1 and not IL-8/CXCR2 inherits this protective effect [28]. Most surprisingly in this context is that the HIV-1 matrix protein p17 binds to both, CXCR1 and CXCR2 in an IL-8-like manner [29]. Taken together, the higher expression of CXCR2 in the wild type group might have implications for the p17_HIV-1_/IL-8 pathway of CXCR1 induced CCR5 internalization resulting in an altered susceptibility.

**Table 3:** Three genes with a link to HIV-1 pathogenesis were found to be significant higher expressed in the CCR5 wild type group

**Significant higher Expression in CCR5 wild type samples**				
Gene	Function *(link to HIV-1)*	Beneficial effect for CCR5-delta32 carriers		Reference
		Transm.	Repli.	
TOP1	Synthesis of cDNA *(in combination with RT transcription of viral RNA to DNA)*		Possible	[30]
CXCR2	Receptor for IL-8. Mediates neutrophil migration to sites of inflammation. *(binding side of p17*_*HIV-1*_*, cross-linked to CXCR1 and indirectly cross-linked to CXCR1 mediated CCR5 internalisation)*	Possible		[29]
SREBF2	Cholesterol metabolism *(enhancing viral replication)*		Possible	[31]

For TOP1 and SREBF2 there are reports that overexpression of these genes might have a beneficial effect on HIV-1 infection. For CXCR2 overexpression this beneficial effect was described in case of HIV-1 transmission. (transm. = transmission, repli. = replication).

Topoisomerase 1 (TOP1) is a 91 kDa enzyme that critically regulates the topological state of DNA during transcription. TOP1 in association with reverse transcriptase is most relevant for HIV cDNA synthesis [32]. Moreover, TOP1 is essential to directly interact with HIV-1 nucleocapsid protein [33] In vitro assays with overexpression of TOP1 led to an 5fold increase of infective virions derived from these manipulated cells [34]. Our finding that TOP1 was significantly underexpressed in CCR5-delta32 samples than in wild type samples could contribute to the clinical observation of decreased rate of progression to AIDS in HIV-1-infected heterozygous carriers.

Finally, cholesterol is known to play an essential role in the life cycle of several enveloped viruses. Many of these viruses manipulate host cholesterol metabolism to facilitate their replication. The target gene of the sterol regulatory element-binding protein 2 (SREBP2) which was found to be downregulated in the heterozygous CCR5-delta32 group is TFII-I, a gene critical for HIV-1 transcription in activated T cells [31]. Experimental knockdown of SREBP2 expression by small interfering RNA (siRNA) resulted in a significant reduced viral replication. Consecutively, decreased expression of SREBP2 in CCR5-delta32 heterozygotes may contribute to benefits of viral replication and progression to AIDS.

### Genes with higher expression in the CCR5-delta32 group

In the CCR5-del32 group, 96 genes have been found to be overexpressed and four of them had a link to HIV-1 infection (Table 4).

**Table 4:** Four genes were found with link to HIV-1 in the CCR5-delta32 heterozygous group

**Significant higher Expression in CCR5-delta32 samples**				
Gene	Function *(link to HIV-1)*	Beneficial effect for CCR5-delta32 carriers		Reference
		Transm.	Repli.	
U2AF65	Splicing factor *(enhances with the polypyrimidine tract of the splicosome of several HIV-1 genes)*	Unknown		[35]
CD30L	Regulation immune response *(elevated CD30 levels during acute HIV-1 infection)*	Unknown		[36]
PRKACB	Catalytic subunit of camp-dependent protein kinase *(coexpression with the HDF GliPR)*	Unknown		[37]
TAP	Antigen presentation *(HIV-1 abrogates TAP mediated peptide transport to MHC I presentation)*	Possible		[38]

Only for TAP there was a beneficial effect pre-described in terms of viral transmission. For the other three genes there are currently no data concerning a beneficial effect available. (transm. = transmission, repli. = replication).

PRKACB was significantly higher expressed in the CCR5-delta32 group. PRKACB is one of five human C subunits of the PKA group which acts as a serine/threonine kinase but only PRKACB together with PRKACA and PRKX do act as functional protein kinases [39]. Concerning HIV-1 pathogenesis this kinase has been described to be involved in infection such as activating viral transcription of monocytes/macrophages in vitro, or activation of T-cells in HIV-patients with ongoing HAART [40,41]. Most interestingly is the transcriptional regulation of CCR5 expression through a cAMP/PKA/CREB dependent pathway. [42] The CCR5 promoter region contains an cyclic AMP responsive element which could be critical for the regulation of gene expression [43]. Although authors could show that a temporal increase of CCR5 expression could be mediated by PKA, the role of differentially regulated PRKACB in HIV-1 pathogenesis still needs to be further determined.

One possible mechanism of HIV-1 escape from immune recognition and consecutive cytolysis is downregulation of the MHC I expression. TAP as a transporter for antigen presentation was overexpressed in the CCR5-delta32 carrier group. It is known that HIV-1 infection abolishes the ability of cells to translocate antigenic peptides via TAP to the endoplasmatic reticulum and thus indirectly abrogates peptides from MHC I presentation [38]. Overexpression of TAP in heterozygotes might partially compensate this HIV-induced inhibition leading to an enhanced clearance of infected cells and indirectly to a decreased susceptibility in this group.

U2AF^65^ is a 65 kDa auxiliary factor of pre-mRNA splicing and contains a sequence-specific RNA-binding region with 3′ RNA recognition motifs and an Arg/Ser-rich domain necessary for splicing. The spliced mRNA species in HIV-1 infected cells are *env/vpu*, *nef*, *rev*, *vpr*, and *vif*. The binding of a 35 kDa subunit stabilizes the binding of U2AF^65^ with the polypyrimidine tract (PPT) of introns during spliceosome assembly [35,44]. However, there are no reports that altered expression of U2AF^65^ contributes to HIV-1 viral progression or susceptibility of transmission.

CD30L is mainly expressed in high levels on activated T cells interacting either with membrane bound CD30 from B or T lymphocytes or soluble CD30. Soluble CD30 has been described to be expressed in high amounts during acute HIV-1 infection [36]. Recently, it has been reported that specified CD4 and CD8 T cell clones are capable of producing T helper (Th)2-type cytokines, which release soluble CD30 into the circulation [45]. The general function of the CD30/CD30L interaction is still unsolved. It has been suggested that this interaction promotes secondary humoral immune responses. However, an in vivo test system with CD30-/- knockout mice or anti CD30L antibodies have led to conflicting results [46]. Taken together, there is no convincing explanation whether the CD30L overexpression in CCR5-delta32 heterozygotes may contribute to a potential benefit in these patients or not.

## Conclusions

We identified 7 genes previously found to be associated with HIV-1 pathogenesis and furthermore were differentially expressed in CCR5 wild type and CCR5-delta32 heterozygous individuals. Out of these two, CXCR2 and TAP may have a beneficial effect concerning HIV-1 transmission, and two genes, TOP1 and SREBF2, may be favorable in terms of replication and disease progression in heterozygous CCR5-delta32 carriers. Concerning the other three genes, there was no evidence of a beneficial effect described so far. The clinical relevance of these findings should be further tested in prospective studies.

## Methods

### Specimen

Input material for this analysis were immunomagnetically purified CD34+ cells from bone marrow aspiration of 19 healthy volunteers, 9 male 10 female, aged 19–85 (median 25) years. All donors gave written informed consent before investigation. Prior to CD34+ selection, mononuclear cells were isolated by density gradient centrifugation through Ficoll-Hypaque (Biochrom, Berlin, Germany). The study protocol was approved by the Ethical committee II, Medical Faculty Mannheim, Heidelberg University Institutional Review Board.

### CCR5 genotyping analysis

Genomic DNA was extracted from heparinised peripheral blood monocytes (PBMC) of the donors with the QIAGEN-Blood-Midi-Kit (Qiagen, Germany). Screening of the donors for the CCR5-delta32-allele was performed with a genomic PCR using primers flanking the site of the deletion (forward: 5′-CTCCCAGGAATCATCTTTACC-3′, reverse: 5′-TCATTTCGACACCGAAGCAG-3′) leading to a PCR fragment of 200 base pairs (bp) for the CCR5-allele and of 168 bp in case of a delta32 deletion. Results were confirmed by allele specific PCR and by direct sequencing using the BigDye^®^-Terminator-1.1.-Cycle-Sequencing-Kit (Applied Biosystems, Germany). Sequences were analyzed using the Vector-NTI-Contig-Express-software (Invitrogen, Germany).

### RNA preparation and array based gene expression analysis

Total RNA was extracted from purified CD34+ hematopoietic progenitor cells using TRIzol (Invitrogen, Karlsruhe, Germany) according to the manufacturer’s protocol. The quality of RNA was determined by the 2100 Bioanalyzer system (Agilent Technologies, Waldbronn, Germany) and only samples showing no RNA-degradation were included into the analysis. Oligonucleotide microarrays (HG-U133plus 2.0, Affymetrix Inc., Santa Clara, CA) were hybridized as described previously [47]. Data analysis was performed by the Microarray Suite 5.0 (Affymetrix), and the Genespring software 4.2 (Silicon Genetics, Redwood City, CA). The quality control parameters were in accordance to the MIAME consensus criteria for micro array data with a present call rate of at least 25% [48]. Previously, our group has shown a close correlation of micro-array data compared to quantitative PCR methods in the same sample [49].

### Statistics

All samples were normalized with expression values raised to an arbitrary value of 1. Only expression values which reach a present call rate of 75% have been used. Only genes with a significant three-fold difference of expression (p < 0.05) between the CCR5 wild type and the CCR5-delta32 group were eligible for further statistical analysis. Expression analysis of the different groups was performed by using the Mann Whitney test.

## Competing interests

The authors confirm that this article content has no conflicts of interest.

## Authors’ contributions

GH designed to study and wrote the manuscript, CB performed CCR5 genotyping, MN analyzed micro array data, DN performed the micro array, MR and HK Contributed to conception and design of the study and interpretation of the data. Revised the manuscript critically for important intellectual content. All authors read and approved the final manuscript.

## Supplementary Material

Additional file 1: Table S1 Results of the Affymetrix Micro Array Assay. Only expressions with 3fold difference, present call rate of 75%, and p < 0.05 have been noted. Click here for file
