# Supplementary material for: Coregulation of HIV-1 dependency factors in individuals heterozygous to the CCR5-delta32 deletion
Source: AIDS Res Ther. 2013 Nov 18;10:26. doi: 10.1186/1742-6405-10-26 (PMC3834523; doi:10.1186/1742-6405-10-26)
Supplement: Additional file 1: Table S1 — Results of the Affymetrix Micro Array Assay. Only expressions with 3fold difference, present call rate of 75%, and p < 0.05 have been noted. [file 1742-6405-10-26-S1.doc]

|  | | CCR5-delta32  heterozygous | CCR5 wild type |  |
| --- | --- | --- | --- | --- |
| Systematic name | Ratio | Normalized | Normalized | Common names, synonyms |
| 208602_x_at | 7,90 | 6,23 | 0,79 | TP120 |
| 211893_x_at | 6,07 | 4,75 | 0,78 | CD6 |
| 1559882_at | 5,52 | 5,63 | 1,02 |  |
| 234664_at | 5,40 | 8,40 | 1,55 |  |
| 1567628_at | 5,36 | 5,60 | 1,04 |  |
| 211789_s_at | 5,13 | 3,67 | 0,72 | MIR, KIAA0867 |
| 227057_at | 4,70 | 3,34 | 0,71 |  |
| 233573_s_at | 4,66 | 4,44 | 0,95 | FLJ10218 |
| 218382_s_at | 4,64 | 5,57 | 1,20 | U2AF65 |
| 221628_s_at | 4,57 | 6,89 | 1,51 | N-PAC |
| 211900_x_at | 4,55 | 4,27 | 0,94 | CD6 |
| 1565599_at | 4,52 | 6,18 | 1,37 |  |
| 206582_s_at | 4,51 | 3,87 | 0,86 | TM7LN4, TM7XN1 |
| 214551_s_at | 4,37 | 4,64 | 1,06 | GP40, TP41, Tp40, LEU-9 |
| 216926_s_at | 4,35 | 3,48 | 0,80 | KIAA0892 |
| 207425_s_at | 4,29 | 5,64 | 1,31 | MSF1, SEPT9, PNUTL4, AF17q25, KIAA0991 |
| 222501_s_at | 4,27 | 4,81 | 1,13 | RIP60 |
| 216850_at | 4,24 | 4,42 | 1,04 | Homo sapiens RT-LI mRNA, complete sequence |
| 233207_at | 4,19 | 3,34 | 0,80 |  |
| 211823_s_at | 4,13 | 6,72 | 1,63 | PXN |
| 230609_at | 4,02 | 14,98 | 3,73 | KIAA0171 |
| 243641_at | 4,02 | 2,84 | 0,71 |  |
| 205095_s_at | 3,93 | 3,46 | 0,88 | VPP1, ATP6N1 |
| 230831_at | 3,84 | 3,19 | 0,83 |  |
| 213606_s_at | 3,84 | 6,30 | 1,64 | ARHGDIA |
| 208442_s_at | 3,83 | 4,50 | 1,18 | AT1, ATA, ATC, ATD, ATDC |
| 202046_s_at | 3,78 | 2,45 | 0,65 | GRF-1, P190A, P190-A, KIAA1722, MGC10745 |
| 234939_s_at | 3,70 | 5,02 | 1,36 | KIAA1523 |
| 215570_s_at | 3,70 | 2,32 | 0,63 | Homo sapiens chromosome 19, cosmid F23269 |
| 231173_at | 3,65 | 2,94 | 0,81 |  |
| 214738_s_at | 3,62 | 3,71 | 1,02 | MGC16714 |
| 207858_s_at | 3,60 | 2,67 | 0,74 | PK1 |
| 201367_s_at | 3,59 | 2,37 | 0,66 | ZFP36L2 |
| 203809_s_at | 3,58 | 3,62 | 1,01 | PRKBB, PKBBETA, RAC-BETA |
| 236310_at | 3,55 | 2,66 | 0,75 |  |
| 1565597_at | 3,53 | 2,50 | 0,71 |  |
| 1566253_at | 3,53 | 3,04 | 0,86 |  |
| 231979_at | 3,52 | 3,59 | 1,02 |  |
| 211571_s_at | 3,50 | 2,79 | 0,80 | CSPG2 |
| 201167_x_at | 3,49 | 3,93 | 1,13 | GDIA1 |
| 215262_at | 3,47 | 3,68 | 1,06 | Homo sapiens clone 24629 mRNA sequence |
| 201008_s_at | 3,46 | 2,99 | 0,87 | TXNIP |
| 1553685_s_at | 3,44 | 5,00 | 1,46 |  |
| 211791_s_at | 3,43 | 2,63 | 0,77 | AKR6A5, KCNA2B, HKVBETA2.1, HKVBETA2.2 |
| 213979_s_at | 3,41 | 3,15 | 0,93 | CTBP1 |
| 215581_s_at | 3,39 | 3,82 | 1,13 | Homo sapiens cDNA FLJ12241 fis, clone MAMMA1001274 |
| 201308_s_at | 3,39 | 2,49 | 0,74 | FLJ10849 |
| 1553691_at | 3,38 | 3,13 | 0,93 |  |
| 1558965_at | 3,37 | 3,03 | 0,90 |  |
| 1555632_at | 3,33 | 3,72 | 1,12 |  |
| 211272_s_at | 3,32 | 3,03 | 0,91 | DGKA |
| 1565716_at | 3,31 | 3,36 | 1,01 |  |
| 238699_s_at | 3,31 | 2,29 | 0,69 |  |
| 232279_at | 3,29 | 1,87 | 0,57 | KIAA0239 |
| 201971_s_at | 3,29 | 3,93 | 1,20 | ATP6A1 |
| 216491_x_at | 3,28 | 2,49 | 0,76 | V4-4 |
| 205179_s_at | 3,25 | 2,41 | 0,74 | ADAM8 |
| 1558937_s_at | 3,25 | 3,56 | 1,10 |  |
| 1553267_a_at | 3,24 | 2,35 | 0,72 |  |
| 209010_s_at | 3,21 | 2,50 | 0,78 | TRIO |
| 221837_at | 3,21 | 2,66 | 0,83 | FLJ14360 |
| 201638_s_at | 3,20 | 2,21 | 0,69 | CPSF1 |
| 214975_s_at | 3,20 | 3,42 | 1,07 | Homo sapiens cDNA FLJ10954 fis, clone PLACE1000383, highly similar to Homo sapiens mRNA for MTMR1 protein |
| 210317_s_at | 3,19 | 2,34 | 0,73 | YWHAE |
| 201282_at | 3,19 | 2,16 | 0,68 | OGDH |
| 223131_s_at | 3,18 | 2,40 | 0,75 | RNF27 |
| 206220_s_at | 3,18 | 2,25 | 0,71 | RASA3, GAPIII |
| 224231_at | 3,15 | 3,49 | 1,11 |  |
| 215646_s_at | 3,15 | 2,59 | 0,82 |  |
| 244055_at | 3,13 | 2,40 | 0,77 |  |
| 239296_at | 3,13 | 2,78 | 0,89 |  |
| 1554152_a_at | 3,11 | 4,64 | 1,49 |  |
| 243054_at | 3,11 | 2,05 | 0,66 |  |
| 243042_at | 3,11 | 2,98 | 0,96 |  |
| 236221_at | 3,10 | 2,17 | 0,70 |  |
| 216901_s_at | 3,10 | 3,52 | 1,14 | IK1, LYF1, LyF-1, hIk-1, IKAROS |
| 211513_s_at | 3,09 | 2,54 | 0,82 | OGFR |
| 214971_s_at | 3,08 | 2,69 | 0,87 | SIAT1 |
| 217859_s_at | 3,07 | 2,47 | 0,80 | FLJ11274 |
| 214251_s_at | 3,07 | 2,00 | 0,65 | NUMA1 |
| 242599_at | 3,06 | 2,23 | 0,73 |  |
| 1552516_a_at | 3,06 | 4,11 | 1,34 |  |
| 240703_s_at | 3,06 | 3,23 | 1,06 |  |
| 213756_s_at | 3,05 | 2,78 | 0,91 | HSF1 |
| 215144_at | 3,05 | 2,17 | 0,71 | Homo sapiens clone 23712 mRNA sequence |
| 209727_at | 3,05 | 2,43 | 0,80 | GM2A |
| 232490_s_at | 3,04 | 2,52 | 0,83 | HTCD37 |
| 1558214_s_at | 3,04 | 1,89 | 0,62 |  |
| 207824_s_at | 3,04 | 2,42 | 0,80 | PUR1, ZF87 |
| 207216_at | 3,04 | 2,18 | 0,72 | CD30L, CD30LG |
| 1560353_at | 3,03 | 2,93 | 0,97 |  |
| 1554273_a_at | 3,03 | 2,73 | 0,90 |  |
| 203676_at | 3,01 | 3,08 | 1,03 | GNS |
| 1568449_at | 3,00 | 2,70 | 0,90 |  |
| 221602_s_at | 3,00 | 2,63 | 0,88 | TOSO |
| 213121_at | 3,00 | 2,34 | 0,78 | SNRP70 |
| 227510_x_at | -3,98 | 0,44 | 1,74 | HDAC3 |
| 216398_at | -3,92 | 0,64 | 2,49 | GYPB |
| 208576_s_at | -3,65 | 0,60 | 2,20 | H3/l |
| 222439_s_at | -3,29 | 0,33 | 1,10 | TRAP150 |
| 233387_s_at | -3,24 | 0,68 | 2,19 |  |
| 226675_s_at | -3,19 | 0,34 | 1,08 | HDAC3 |
| 232556_at | -3,17 | 0,57 | 1,82 |  |
| 228582_x_at | -3,07 | 0,83 | 2,54 | PRO1073 |
| 235244_at | -3,06 | 0,48 | 1,48 |  |
| 208506_at | -3,05 | 0,65 | 1,97 | H3/i |
| 240602_at | -3,02 | 0,38 | 1,16 |  |
| 231616_at | -2,91 | 0,73 | 2,13 |  |
| 204848_x_at | -2,81 | 0,71 | 2,00 | HBGA |
| 202581_at | -2,80 | 0,62 | 1,74 | HSP70-2 |
| 243032_at | -2,77 | 0,60 | 1,67 |  |
| 208900_s_at | -2,75 | 0,39 | 1,07 | TOP1 |
| 210119_at | -2,75 | 0,58 | 1,61 | KCNJ15 |
| 224567_x_at | -2,70 | 0,40 | 1,09 | HDAC3 |
| 208553_at | -2,69 | 0,52 | 1,41 | H1.4 |
| 204419_x_at | -2,69 | 0,76 | 2,05 | HBG2 |
| 223796_at | -2,68 | 0,70 | 1,88 | FLJ14195, KIAA1714 |
| 241955_at | -2,66 | 0,52 | 1,39 |  |
| 204415_at | -2,66 | 0,63 | 1,69 | 6-16, IFI616 |
| 1561058_at | -2,59 | 0,65 | 1,70 |  |
| 1562056_at | -2,57 | 0,52 | 1,34 |  |
| 211434_s_at | -2,56 | 0,82 | 2,10 | HCR, CKRX, CRAM-A, CRAM-B |
| 241205_at | -2,53 | 0,77 | 1,95 |  |
| 223940_x_at | -2,52 | 0,55 | 1,39 | HDAC3 |
| 224568_x_at | -2,51 | 0,49 | 1,24 | HDAC3 |
| 1559129_a_at | -2,51 | 0,64 | 1,60 |  |
| 219334_s_at | -2,50 | 0,67 | 1,69 | FLJ13624 |
| 229548_at | -2,49 | 0,50 | 1,24 | KIAA0668 |
| 204909_at | -2,49 | 1,06 | 2,65 | P54, RCK, HLR2 |
| 217878_s_at | -2,47 | 0,52 | 1,29 | CDC27 |
| 230738_at | -2,47 | 0,51 | 1,27 |  |
| 207459_x_at | -2,47 | 0,64 | 1,57 | SS, MNS |
| 203502_at | -2,43 | 0,71 | 1,72 | BPGM |
| 243424_at | -2,42 | 0,65 | 1,58 |  |
| 243259_at | -2,41 | 0,69 | 1,67 |  |
| 235009_at | -2,40 | 0,63 | 1,51 |  |
| 35201_at | -2,38 | 0,73 | 1,75 | HNRPL |
| 236665_at | -2,38 | 0,62 | 1,48 |  |
| 242496_at | -2,37 | 0,74 | 1,75 |  |
| 235354_s_at | -2,37 | 0,72 | 1,71 | LOC51319 |
| 1560705_at | -2,35 | 0,58 | 1,38 |  |
| 222837_s_at | -2,31 | 0,76 | 1,75 | NATH |
| 204840_s_at | -2,31 | 0,59 | 1,36 | EEA1 |
| 209398_at | -2,30 | 0,54 | 1,25 | H1.2 |
| 205592_at | -2,30 | 0,70 | 1,61 | SLC4A1 |
| 215635_at | -2,29 | 0,69 | 1,57 | Homo sapiens mRNA full length insert cDNA clone EUROIMAGE 239714 |
| 215078_at | -2,29 | 0,94 | 2,15 | Homo sapiens mRNA; cDNA DKFZp564M2422 (from clone DKFZp564M2422); partial cds |
| 206515_at | -2,29 | 0,71 | 1,61 | CPF3, CYP4F, LTB4H |
| 204007_at | -2,29 | 0,71 | 1,63 | FCGR3B |
| 239923_at | -2,29 | 0,62 | 1,42 |  |
| 213515_x_at | -2,28 | 0,77 | 1,76 | MYL4 |
| 229967_at | -2,27 | 0,81 | 1,84 |  |
| 240108_at | -2,26 | 0,65 | 1,47 |  |
| 209906_at | -2,26 | 0,72 | 1,62 | AZ3B, C3AR, HNFAG09 |
| 1558710_at | -2,25 | 0,64 | 1,45 |  |
| 1557529_at | -2,24 | 0,81 | 1,83 |  |
| 202072_at | -2,24 | 0,78 | 1,75 | HNRPL |
| 223527_s_at | -2,23 | 0,86 | 1,91 | NYD-SP15 |
| 233233_at | -2,23 | 0,71 | 1,59 |  |
| 202203_s_at | -2,22 | 0,54 | 1,20 | GP78 |
| 210429_at | -2,21 | 0,67 | 1,49 | RH, RhII, RhPI, DIIIC, DIIIc, RHDVA(TT), RhDVa(TT) |
| 206108_s_at | -2,21 | 0,65 | 1,43 | SFRS6 |
| 230026_at | -2,20 | 0,75 | 1,64 | MRPL43 |
| 204141_at | -2,20 | 1,00 | 2,20 | TUBB |
| 223674_s_at | -2,19 | 0,75 | 1,64 | SPEC1 |
| 227341_at | -2,19 | 0,55 | 1,21 |  |
| 204466_s_at | -2,18 | 0,72 | 1,56 | SNCA |
| 236793_at | -2,17 | 0,77 | 1,66 |  |
| 230805_at | -2,17 | 0,68 | 1,47 | SREBF2 |
| 228214_at | -2,16 | 0,64 | 1,37 |  |
| 1556923_at | -2,15 | 0,96 | 2,06 |  |
| 240843_at | -2,14 | 0,67 | 1,44 |  |
| 238548_at | -2,14 | 0,65 | 1,38 |  |
| 208859_s_at | -2,13 | 0,57 | 1,22 | ATRX |
| 214465_at | -2,12 | 0,68 | 1,45 | ORM2 |
| 229457_at | -2,12 | 0,59 | 1,24 |  |
| 235476_at | -2,10 | 0,86 | 1,80 |  |
| 241616_at | -2,10 | 0,63 | 1,33 |  |
| 224316_at | -2,10 | 0,75 | 1,57 | FLJ20038 |
| 222294_s_at | -2,10 | 0,66 | 1,38 | ESTs |
| 236081_at | -2,10 | 0,74 | 1,56 |  |
| 1558695_at | -2,09 | 0,54 | 1,12 |  |
| 214464_at | -2,09 | 0,66 | 1,39 | PK428 |
| 217427_s_at | -2,08 | 0,59 | 1,23 | TUP1, DGCR1, TUPLE1 |
| 1554411_at | -2,08 | 0,61 | 1,27 |  |
| 234701_at | -2,07 | 0,61 | 1,27 | LZ16 |
| 242772_x_at | -2,07 | 0,76 | 1,57 |  |
| 204970_s_at | -2,07 | 0,56 | 1,16 | MAFG |
| 228910_at | -2,06 | 0,54 | 1,12 | KAI1 |
| 236291_at | -2,05 | 0,69 | 1,41 |  |
| 1560758_at | -2,05 | 0,72 | 1,48 |  |
| 228648_at | -2,05 | 0,59 | 1,22 | LRG |
| 209258_s_at | -2,04 | 0,64 | 1,30 | BAM, HCAP, SMC3, SMC3L1 |
| 216594_x_at | -2,04 | 0,91 | 1,85 | AKR1C1 |
| 1558678_s_at | -2,04 | 0,61 | 1,24 |  |
| 207008_at | -2,03 | 0,81 | 1,64 | CXCR2, IL8RA, CMKAR2 |
| 225820_at | -2,03 | 0,55 | 1,12 |  |
| 236446_at | -2,03 | 0,69 | 1,39 |  |
| 209890_at | -2,03 | 0,71 | 1,43 | NET-4 |
| 200947_s_at | -2,03 | 0,78 | 1,59 | GLUD1 |
| 230972_at | -2,02 | 0,80 | 1,61 |  |
| 204151_x_at | -2,02 | 0,79 | 1,59 | AKR1C1 |
| 210511_s_at | -2,02 | 0,66 | 1,34 | INHBA |
| 210586_x_at | -2,02 | 0,72 | 1,46 | RHCE |
| 239196_at | -2,02 | 0,65 | 1,32 |  |

Supplemental Table 1.

Results of the Affymetrix Micro Array Assay. Only expressions with 3fold difference, present call rate of 75%, and p< 0.05 have been noted.
